# Expression and clinical significance of PD-L1 and BRAF expression in nasopharyngeal carcinoma

**DOI:** 10.1186/s12885-019-6276-y

**Published:** 2019-10-29

**Authors:** Yabing Cao, Kin Iong Chan, Gungli Xiao, Yanqun Chen, Xibin Qiu, Hu Hao, Sao Chi Mak, Tongyu Lin

**Affiliations:** 1 Department of Oncology, Kiang Wu Hospital, Macau, SAR China; 2 Department of Pathology, Kiang Wu Hospital, Macau, SAR China; 3 Department of Oncology, Sun Yat-Sen University Cancer Center, Guangzhou, China

**Keywords:** Nasopharyngeal carcinoma, Programmed death-ligand 1, BRAF, Prognosis

## Abstract

**Background:**

The prognostic value of programmed death-ligand 1 (PD-L1) and BRAF expression in nasopharyngeal carcinoma (NPC) is not well-defined. In this study we investigated alterations in PD-L1, BRAF and EGFR by using immunohistochemistry analysis in a cohort of consecutively enrolled NPC patients.

**Methods:**

A retrospective review of 154 NPC patients form our previous study (BMC Cancer. 2013; 13:226) were conducted. Survival and prognostic impacts were analyzed based on PD-L1, BRAF and EGFR expression levels.

**Results:**

One hundred fifty four patients were included in this study. PD-L1 expression was detected in 87.7% of patients; 14.3% had 1–5% PD-L1 expression, 47.4% had 5–49% expression while 26% had ≥50% expression Higher PD-L1 expression was significantly associated with shorter PFS and OS. The median PFS was 25 months (95% CI 15.7–34.3 months) and OS was 35 months (95% CI 22.60–47.4 months) for patients with PD-L1 expression ≥50%; both median PFS and OS were not yet reached for patients with PD-L1 expression < 50%. PFS was significantly higher in BRAF mutation positive patients (5-year PFS: 55.1% vs. 30.8%, *P* = 0.044).

**Conclusion:**

Tumor PD-L1 expression and BRAF mutation are associated with poor outcomes in patients with NPC. This study was retrospectively registered in ClinicalTrials.gov (NCT03989297) on 2019-6-18.

## Background

Nasopharyngeal carcinoma (NPC) is rare in most parts of the world but is one of the more common types of cancer in southern China. In 2015, it was estimated that the incidence of NPC was 60.6 per 100,000 in China with a mortality rate of 34.1 per 100,000 [1, 2]. The main treatment for NPC is radiotherapy or chemoradiotherapy [3], and the 5-year survival rate is about 85% [4]. Even with best available treatment, about 30% of patients relapse with local recurrence or metastasis [5]. The prognosis for patients with recurrent or primary metastatic NPC is poor with a median progression free survival of 19.4 months [6]. Evidently, novel approaches and better therapies are needed for the treatment of NPC.

Biomarkers that can reliably predict the prognosis of patients are important. In a previous study, we found that gender and age were strong independent prognostic factors for NPC [7]. Specifically, younger and male patients were more likely to have distant metastases and exhibit poorer overall survival and progression-free survival rates compared to other NPC patients treated in our center [7]. A more recent study identified a prognostic gene expression-based signature that predicts distant metastasis in locoregionally advanced NPC [8].

In addition to prognostic biomarkers, predictive biomarkers that can identify patients who are likely to benefit from a particular therapy can help guide treatment selection. NPC is characterized by lymphocyte infiltration, including T cells and cytotoxic tumor-infiltrating T lymphocytes [9]. Since immune checkpoint inhibitors can activate cytotoxic T cells to attack cancer cells, patients with lymphocyte-rich cancer types (such as EBV-positive NPC) may benefit more from immunotherapy [10, 11]. Tumor programmed death-ligand 1 (PD-L1) expression levels have also been suggested to be of predictive value for treatment efficacy in some cancer types [12–15]. However, the clinical significance of PD-L1 expression in NPC is controversial due to conflicting data amongst studies [16–19].

BRAF is one of downstream of EGFR pathway molecule [20], and BRAF (V600E) mutation is rarely reported in previous study [21]. In other solid tumors such as melanoma and non-small cell lung cancer, BRAF inhibitors were approved for patients with BRAF mutation positive.

In the present study we aim to evaluate the clinical significance of PD-L1, BARF and EGFR expressions in the tumor cells of a cohort of NPC patients. Separate data from this cohort of patients have been reported in a previous publication [7].

## Methods

### Patient selection

Consecutive patients who were pathologically diagnosed with NPC between 2006 and December 2010 at the Kiang Wu Hospital (Macau SAR of China) and for whom fresh-frozen tissue samples were available were included. The clinicopathologic information of all patients was collected, including sex, age, tumor stage, pathologic type, and treatment methods and outcomes. Tumor stage was classified according to the International Union Against Cancer and American Joint Committee on Cancer staging system for NPC, seventh edition. Fresh nasopharyngeal tissue samples were obtained from all patients. The protocol was approved by the institutional review board of the Kiang Wu Hospital (KWH 2016–014).

### Treatment and outcome

All patients received standard treatment including radiation therapy with or without chemotherapy. Briefly, the intensity modulated radiotherapy technique technology were utilized for radiation. Chemotherapy were given for patients based on their tumor stage and the decision by each patient’s physician. Chemotherapy regimen was based on NCCN guidelines.

We defined progression-free survival (PFS) as time from date of treatment to the date of disease progression or death from any causes, whichever came first. Overall survival (OS) was defined as the time from date of treatment to the time of death.

### Immunohistochemistry for PD-L1, BRAF, and EGFR expression

PD-L1, BRAF and EGFR expressions in the tumor cells was evaluated using immunohistochemistry. Four mm-thick sections were prepared from paraffin-embedded specimens of the NPC tumor. The sections were deparaffinized in xylene followed by 95% ethanol. After rehydration, sections were pretreated in a microwave oven at 95 °C for 15 min in citrate buffer (pH 6.0) for antigen retrieval. Next, endogenous peroxidase activity was blocked with 4% Block ACE Powder in H2O at 37 °C for 10 min.

### Immunohistochemistry (IHC) was carried out by benchmark XT automated stainer

PD-L1 protein was detected by using PD-L1 (SP263) rabbit monoclonal antibody with Ultraview detection system (Ventana, Tucson, Arizona). Reference to the interpretation guide of Ventana PD-L1 (SP263) assay staining of non-small cell lung cancer, the tumor cells was counted if any intensity of the staining result demonstrating in membrane with a discontinuous, circumferential or basolateral pattern or rarely in peri-nuclear dot-like body.

BRAF V600E protein was detected by using BRAF V600E (VE1) mouse monoclonal primary antibody and the OptiView DAB IHC Detection Kit (Ventana, Tucson, Arizona). The immunostaining result was interpreted as positive if any intensity of cytoplasmic staining.

EGFR mutation specific antibodies were detected by using EGFR mutation specific rabbit monoclonal antibodies against del E746-A750 (6B6, dilution:1:50; Cell Signaling Technology, Inc., Boston, MA, USA) and L858R (43B2, dilution:1:10; Cell Signaling Technology, Inc). The immunoreactions were detected by OptiView DAB IHC Detection Kit (Ventana, Tucson, Arizona).

The immunostaining results were interpreted as positive if any intensity on cytoplasmic and/or membrane staining.

### Assessment of PD-L1, BRAF, and EGFR expression

PD-L1, BRAF mutation, and EGFR mutation expression in tumor cells were evaluated in a blind fashion without knowledge of any existing clinical characteristics. Any staining within the tumor cell membrane or cytoplasm was considered positive. Grading was based on staining ratio of the tumor cells, ≥50% of tumor cells positive was scored as 3; ≥5 to < 50% (5–49%) of tumor cells expressed positive as 2; ≥1 to < 5% (1–5%) of tumor cells expressed positive as 1; negative as 0.

BRAF and EGFR mutation expression were categorized as negative or positive.

### Statistical analysis

Fisher’s exact test or the chi-squared test was performed to examine the association between PD-L1 expression and the oncogenic mutations versus various clinicopathological features, as appropriate. The PD-L1 expression was evaluated as a categorical variable (0, 1–5%, 5–49% and ≥ 50% expression). Survival curves were plotted using the Kaplan-Meier method and compared using a log-rank test. The prognostic impact of relevant clinicopathological variables including PD-L1 expression in the pulmonary metastatic tumors was evaluated using the Cox proportional hazards regression models and hazard ratios (HRs). To assess the prognostic value of high PD-L1 expression, variables with *P* < 0.2 in the univariate analysis were entered into the multivariate analysis, and variables with *P* < 0.05 were included in a final model with backward elimination methods. A two-sided *P*-value< 0.05 was considered statistically significant. Statistical analyses were performed using the SPSS version 20.0 software package (SPSS Inc., Chicago, IL, USA).

## Results

### Patient characteristics

A total of 154 patients were included in the analysis. The baseline characteristics of patients are shown in Table 1. Median age was 60 years (range 26–83 years). The majority of patients were male (75.2%). All patients were diagnosed with non-keratinizing undifferentiated carcinoma according to the WHO histological classification. The median and maximum follow-up duration was 76 months and 145 months, respectively. The last day of follow-up was in January 2019. Seventy six patients (49.4%) had tumor recurrence or metastasis. None of the patients received anti-PD-L1 antibody treatment because anti-PD-L1 antibody treatment was not available in Macau during the follow-up period.

**Table 1:** Patient demographics and disease characteristics

Characteristic		*N* = 154	
		Cases	Percentage (%)
Age (Years)			
	Median	60	
	Range	26–83	
	< 60	71	46.1
	≥60	83	53.9
Sex			
	Male	116	75.2
	Female	38	24.8
Stage			
	I–II	67	43.5
	III–IV	86	56.5
Treatment			
	Chemoradiation	124	80.5
	Radiation Only	31	19.5
ECOG			
	0–1	131	85.0
	≥2	24	15.0
Progression			
	Yes	76	49.4
	No	78	50.6
PD-L1			
	0%	17	11.0
	1–5%	22	14.3
	5–49%	73	47.4
	≥50	40	26.0
	Unknown	2	1.3
BRAF V600E			
	Negative	139	90.3
	Positive	13	8.4
	Unknown	2	1.3
EGFR 19del			
	Negative	149	96.8
	Positive	3	1.9
	Unknown	2	1.3
EGFR L858R			
	Negative	151	98.1
	Positive	0	0.0
	Unknown	3	1.9

Values are presented as number (%) unless otherwise stated

Percentages may not sum to exactly 100 due to rounding

### Expression of PD-L1, BRAF and EGFR

Fig. 1

**Fig. 1:**
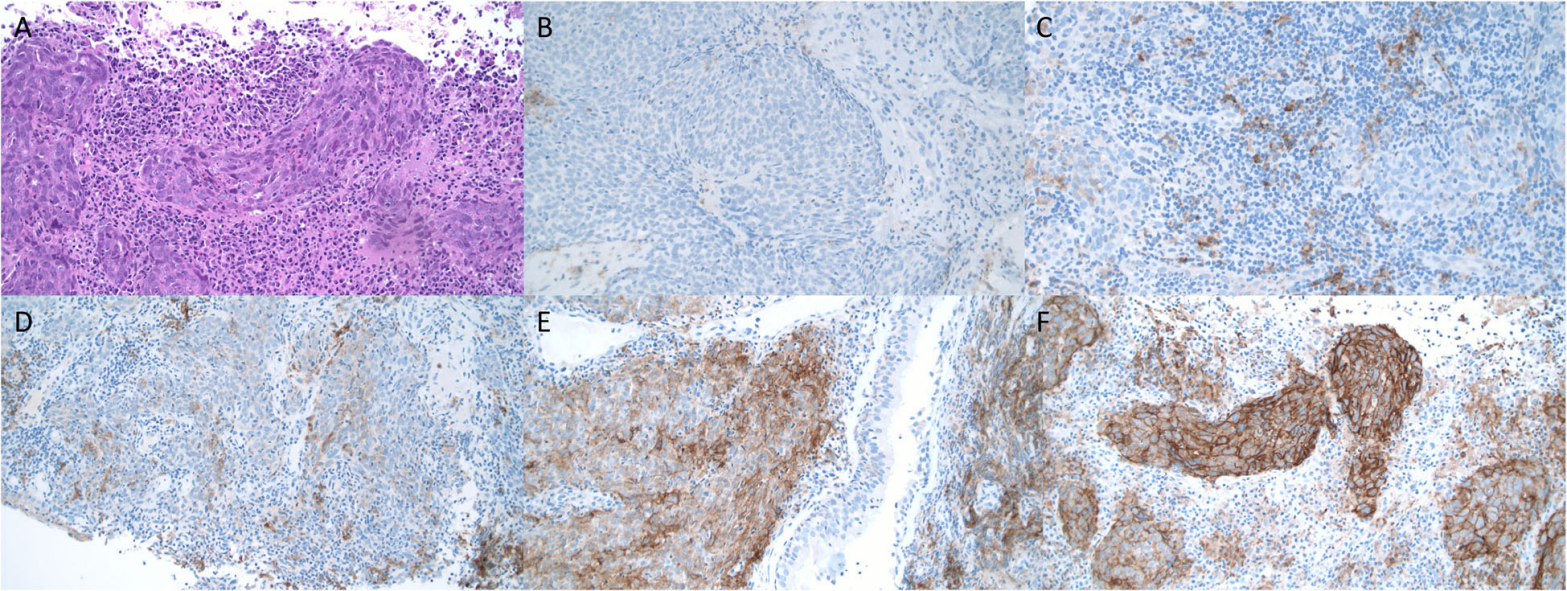
Representative immunostaining of programmed death-ligand 1 (PD-L1), BRAF V600E mutation and EGFR 19del and L858R mutations (magnification, × 200). Anti-PD-L1 antibody (clone SP263) is validated using placenta as a positive control. HE staining of NPC tissue is presented in A. PD-L1 expression in NPC biopsy tissues was graded as 0% (**b**), 1–5% (**c**), 5–49% (**d**), and ≥ 50% (**e** and **f**). BRAF V600E and EGFR staining not shown

### Relationship between PD-L1, BRAF mutation, and EGFR mutation expression with patient characteristics

PD-L1 expression was detected in 87.6% of biopsy tissue. PD-L1 expression was 0% in 11.0% of patients, 1–5% in 14.3% of patients, 5–49% in 47.4% of patients and ≥ 50% in 26% of patients. There was no difference in PD-L1 expression between genders or age groups. However, there was significantly higher expression of PD-L1 among patients with disease recurrence or metastasis (*P* = 0.001). There was also a significantly higher expression of BRAF mutation among patients with disease recurrence or metastasis (*P* = 0.035). There was no significant association between PD-L1 expression levels, BRAF V600E mutation and EGFR 19del mutation with age, sex or disease stage. Most of the tumor tissues that expressed PD-L1 were BRAF V600E mutation negative (*P* = 0.002). There was no significant association between PD-L1 expression levels and EGFR 19del mutation (*P* = 0.161).

### Prognostic impact on progression free survival and overall survival

**Fig. 2:**
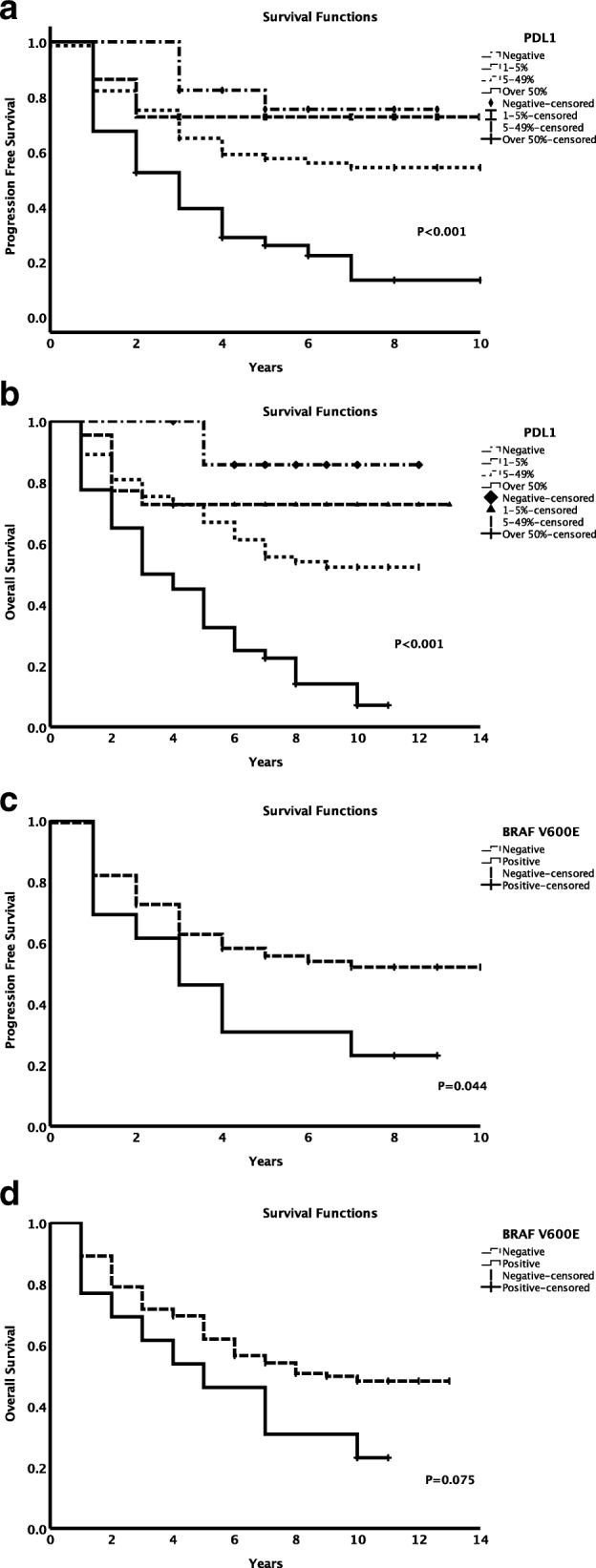
Progression free survival (PFS) and overall survival (OS) for all patients. PFS (**a**) and OS (**b**) by PD-L1 expression levels. PFS (**c**) and OS (**d**) by BRAF V600E mutation

PD-L1 expression was significantly associated with overall survival. Higher expressions of PD-L1 were associated with shorter PFS (*P* < 0.001, Fig. 2a) and reduced OS (P < 0.001, Fig. 2b). The 5-year PFS rates for patients with PD-L1 expression 0%, 1–5%, 5–49% and ≥ 50% were 75.5, 72.7, 55.9 and 24.8%, respectively (P < 0.001). The median PFS was 25 months (95% CI 15.7–34.3 months) for patients with PD-L1 expression ≥50%, and not yet reached for patients with PD-L1 expression < 50%. The overall median PFS for all patients was 84 months. The 5-year OS rate for patients with PD-L1 expression 0%, 1–5%, 5–49% and ≥ 50% were 85.7, 72.7, 68.3 and 35.0%, respectively (*P* < 0.001). The median OS was 35 months (95% CI 22.60–47.4 months) for patients with PD-L1 expression ≥50%, and not yet reached for patients with PD-L1 expression < 50%. The overall median OS for all patients was 96 months (95% CI 60.2–131.8 months). Consistent with our previous report, female patients had a favorable prognosis than male patients (*P* = 0.009, figure not show).

PFS was significantly different between BRAF mutation negative and positive patients (5-year PFS: 55.1% vs. 30.8%, *P* = 0.044; Fig. 2c). However, OS rates did not differ significantly between BRAF mutation negative and positive patients (5-year OS rate: 61.9% vs. 46.2%, *P* = 0.075; Fig. 2d).

**Fig. 3:**
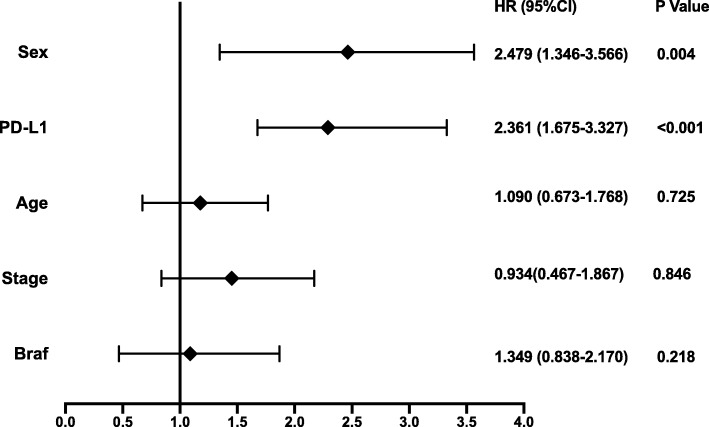
Forest plot of hazard ratio (HR) for overall survival (OS) by independent prognostic factors

The results of prognostic factor analysis for survival using Cox proportional hazards regression model are shown in Fig. 3. Univariate analysis showed that high PD-L1 expression and the female gender were significantly associated with a shorter OS. Multivariate analysis indicated that high PD-L1 expression, along with gender is associated with shorter OS and thus poorer prognosis. There was no interaction between PD-L1 expression and gender (Table 2). The presence of BRAF V600E mutation was associated with disease progression (*P* = 0.035; Table 2).

**Table 2:** Association between clinical parameters and expression of PD-L1, BRAF and EGFR proteins

Characteristic	PD-L1 (N)					BRAF V600E (N)			EGFR 19Del (N)		
	0	1–5	5–49	> 50	P-value	Negative	Positive	P-value	Negative	Positive	P-value
Age											
< 60	10	11	36	12	0.127	64	5	0.411*	67	2	0.431*
≥ 60	7	11	37	28		75	8		82	1	
Sex											
Male	14	17	53	30	0.776	104	10	0.602*	112	2	0.581*
Female	3	5	20	10		35	3		37	1	
Stage											
I-II	7	9	36	14	0.517	60	6	0.529*	65	1	0.599*
III-IV	10	13	37	26		79	7		84	2	
Progression											
Yes	4	6	32	33	0.001	65	10	0.035*	73	2	0.490*
No	13	16	41	7		74	3		76	1	

*: Fisher’s exact test

Chi-squared test was used in variables without *

## Discussion

It is known that PD-L1 expression is upregulated on various tumor cell lines. NPC is an EBV-associated cancer. Previous studies demonstrated that EBV-related latent membrane protein 1 (LMP1) and interferon-gamma (IFN-γ) may upregulate PD-L1 in NPC [22, 23] and NK/T cell lymphoma [24]. Expression of viral proteins, such as EBV nuclear antigen-1 or LMP1 and 2 in NPC cells can elicit a virus-specific immune response in patients with NPC. LMP1 expression and IFN-γ activation can synergistically induce the expression of PD-L1 in NPC cells [22]. Expression of PD-L1 can also be upregulated by tumor-infiltrating lymphocytes (TILs), which is associated with impaired effector function (cytokine production and cytotoxic efficacy against tumor cells) and poor outcomes in NPC [25]. In our study, we found positive PD-L1 expression in 87.7% of patients with NPC; 14.3% had 0–1% PD-L1 expression, 47.4% had 1–49% expression while 26% had ≥50% expression. This is consistent with other studies which reported PD-L1 expression in 89–95% of NPC tumors, with 50% or more malignant cells being PD-L1 positive in the majority of these tumors [26].

Activation of PD-1 pathway can lead to T cell exhaustion. Thus, the PD-1/PD-L1 axis is crucial in regulating anti-tumor immunity. In this study, we performed a retrospective analysis on 154 consecutive patients who were homogeneously treated with IMRT. Our findings demonstrate that high PD-L1 expression is a poor prognostic factor for NPC patients. Best progression free survival was seen in the PD-L1 expression negative group, with a 5-year PFS rate of 75.2%. For patients with positive PD-L1 expression, the PFS rate reduces as expression levels increase; 5-year PFS rates were 72.7, 55.9 and 24.8% for patients with PD-L1 expression 1–5%, 5–49% and ≥ 50%, respectively. The 5-year OS rate for patients with PD-L1 expression 0%, 1–5%, 5–49% and ≥ 50% were 85.7, 72.7, 68.3 and 35.0%, respectively. Our data are consistent to those recently published by Ben-Betzalel et al. [19, 27–29] who found similar association of PD-L1 expression with poor survival. However, other studies have reported favorable prognosis with increased PD-L1 expression [17, 30], while others found no relation between PD-L1 expression and survival [31, 32].

There many reasons behind these inconsistent findings. First, some studies included a mixed patient population, which consists of patients with NPC patients as well as those with other types of head and neck squamous cancer. Second, not all studies used commercially available clones of PD-L1 antibodies. SP263 and 22C3 (Dako), and SP142 (Ventana) have been shown to pass the Western Blot and immunohistochemical validation. In prior comparison trials, it was shown that 22C3 and SP263 were closely aligned in tumor cell staining, but SP142 stains less tumor cells [33]. Third, the follow up period of some of the studies were too short for PFS and OS analysis. Our study focused on NPC patients and with an extended follow-up period of 13 years. Since the percentage of PD-L1-positive cells can vary due to different antibody clones and immunostaining methods, finding the best cutoff value with the highest clinical significance is crucial in such studies. We used the SP263 antibody with the standard cut off value of 1 and 5%, which is frequently used for lung cancer and other cancer types [34]. Inevitably, whenever an IHC-based biomarker is considered, questions will arise regarding the reproducibility of the staining of the tissue and consistency in interpretation of the test by pathologists. In future, multicenter, international standardization efforts could address many of these questions and help develop one “standardized” assay to analyse additional immunotherapy-related predictive markers [35].

BRAF mutations have been identified in melanoma and colorectal cancer, but is rarely reported in NPC [36]. BRAF mutations are associated with poorer survival in patients with melanoma [37], but the significance of BRAF mutations among NPC patients has not been thoroughly investigated. For the first time, we report that the BRAF V600E mutation was significantly associated with disease progression and PFS. In this study, 13 of 154 patients (8.4%) were BRAF V600E mutation positive. The 5-year PFS of BRAF V600E mutation positive and negative patients were 55.1 and 30.8%, respectively.

Using multivariate analysis, PD-L1 expression and gender were independent prognostic factors for overall survival. This confirmed our previous study, that female patients had a favorable prognosis than male patients.

PD-L1 expression is the most extensively studied biomarker with respect to predicting the efficacy of anti–PD-1 or anti–PD-L1 therapies. A positive correlation between PD-L1 expression and treatment efficacy has been reported in the study of nivolumab [38] and pembrolizumab for NPC [39]. In our center, 70 patients with NPC have received PD-1 therapy, 13 received nivolumab monotherapy, 29 received pembrolizumab monotherapy and 28 received pembrolizumab combined with chemotherapy. An internal analysis of these patients revealed that PD-L1 positive tumor cell with high CD8 positive tumor infiltrates correlated with objective response to PD-L1 inhibitor (data not published).

Our study has some limitations. Our study lacks EBV loading data as the EBV DNA test was not routinely carried out during the period that patients received treatment. EBV expression is an import contributor here and may increase PD-l1 expression [24, 40]. Secondly, patients in this study did not receive PD-1 or PD-L1 targeted therapy as these were not available in Macau during the follow-up period. Therefore, we were unable to explore the correlation between PD-L1 expression and the efficacy of immunotherapy.

## Conclusion

Our results suggest that high tumor PD-L1 expression and BRAF V600E mutation are associated with poor outcomes in patients with NPC. PD-L1 expression was found to be a significant prognostic factor, and high PD-L1 expression may be of prognostic value for disease progression and survival.

## Data Availability

The datasets used and analyzed during the current study are available from the corresponding author on reasonable request.

## Abbreviations

PD-L1: programmed death-ligand 1 (PD-L1)
NPC: nasopharyngeal carcinoma (NPC)
EGFR: epidermal growth factor receptor
PFS: progression-free survival
OS: overall survival
IHC: Immunohistochemistry
LMP-1: latent membrane protein 1
EBV: Epstein–Barr virus
